# Preparing for a paradigm shift in aging populations: listen to the oldest old

**DOI:** 10.1080/17482631.2018.1511768

**Published:** 2018-08-29

**Authors:** Hiroko Komatsu, Kaori Yagasaki, Hisashi Kida, Yoko Eguchi, Hidehito Niimura

**Affiliations:** a Faculty of Nursing and Medical Care, Keio University, Tokyo, Japan; b Department of Neuropsychiatry, Keio University School of Medicine, Tokyo, Japan

**Keywords:** Oldest old, aging, centenarians, geriatric care, qualitative research

## Abstract

**Purpose**: Current healthcare systems are not suitable for serving future societies in which the oldest old are commonplace. The objective of this study was to understand what the oldest old care most about in their daily lives.

**Methods**: Semi-structured in-depth interviews and thematic analysis were used. Face-to-face interviews were conducted in 17 elderly residents (≥ 95 years) of Arakawa-ku, Tokyo, Japan from July to November 2017.

**Results**: Three themes emerged from the interview responses: “unshakable beliefs and social ties,” “natural acceptance,” and “my day-to-day life with precious moments.” The oldest old strongly believe in diligence and compassion and maintain strong relationships with people around them. Despite their small social networks, they are concerned about future society. They accept their selves and their lives, including their impending deaths. Despite their functional decline, they control their lives by making very small decisions. They live on a moment-to-moment basis, cherishing simple events.

**Conclusion**: Maintaining autonomy through making small decisions and enjoying small pleasures are important to the oldest old. Understanding the needs of the oldest old is the first step towards developing optimal geriatric care for an aging population.

## Introduction

We are approaching the era where providing routine care for people over 100 years of age is common. Globally, the number of the very elderly is growing faster than any other age group (World Health Organization, 2015), with the proportion of centenarians increasing particularly rapidly, and it is estimated that by 2050 there will be 23.6 centenarians for every 10,000 adults aged 65 and older (United Nations, Department of Economic and Social Affairs, Population Division, 2015). Such a dramatic increase in the number of very old people presents a serious challenge for health and social care systems (Zeng, Feng, Hesketh, Christensen, & Vaupel, 2017).

Overuse of medical care among older populations is receiving increasing scrutiny in terms of safety and costs. Polypharmacy is especially prevalent in care of older people; in developed countries, almost 30% of patients aged 65 years and older are prescribed five or more drugs (Qato et al., 2008). Reasons for this over-prescription include the providers’ belief that more care is better, the providers’ poor knowledge of patient preference, and patients’ desire for reassurance (Morgan et al., 2015). Furthermore, technological medical advancements and physicians’ cure-oriented training have blurred the boundaries of medicine. Consequently, aging and dying have recently become medicalized, which can lead to more harm than benefit for the older population (O’Mahony, 2016; van Dijk, Faber, Tanke, Jeurissen, & Westert, 2016). Thus, the conventional “cure-seeking” model of medical care and the current trend towards medicalization are not suitable for the very elderly, meaning a paradigm shift is urgently required in rapidly aging countries (Arai et al., 2015).

The core components of successful aging include maintaining a high level of mental and physical functioning, minimizing the risk of disease and disability, and ensuring social inclusion (Anderson, Goodman, Holtzman, Posner, & Northridge, 2012). Recent studies have highlighted the impact that psychological factors, “such as having a purpose in life,” have on living longer lives (Cohen, Bavishi, & Rozanski, 2016), in addition to maintaining physical functioning (Kim, Kawachi, Chen, & Kubzansky, 2017). Wong et al. (2014) also reported four themes related to living long and well: positive relations with others, positive events and happiness, hope for the future, and positive life attitude. Jopp et al. (2016) addressed loss of well-liked activities and loss of family members as psychological and social challenges, respectively. However, how best to design and implement initiatives that address the needs and preferences of older adults remain unclear (Lette et al., 2017). The perspectives of the oldest old are crucial for shaping future geriatric care services, but their voices are rarely heard. This study aims to determine what the oldest old care about in their daily lives in Japan, which is at the forefront of aging societies, which can help develop suitable geriatric care for the future.

## Methods

### Study design

We conducted individual interviews in this qualitative study to understand daily living experiences of people aged 95 years and older from individual participants’ perspectives. Thematic analysis is a method to identify, analyze and report patterns (themes) within data (Braun & Clarke, 2006), and therefore we used thematic analysis to determine what the oldest old care about in their daily lives.

### Setting and participants

This study is a part of an oldest-old cohort study of 542 residents who were aged 95 years and older as on 1 January, 2016, from the resident registry of Arakawa-ku, a district of Tokyo. In the original cohort study, a questionnaire was mailed to 542 residents to assess their physical, mental, and social status; however, because of the large size of questionnaire, number of valid responses was 41 after excluding 85 residents who were dead or had moved out from the district. Of the 41, 26 residents from the original cohort study agreed to a home-visit medical examination. However, we excluded eight older adults who were determined by a psychiatrist and a psychologist of the research team to be unable to participate; this included individuals who had poor-health condition or difficulty in communication. A research assistant gave an overview, by telephone, of this qualitative study to these 18 older adults or their family caregivers and asked them to agree to home-visit interviews for this study. After providing an explanation of voluntary participation, we made appointments for home visits with the 18 participants, some including their caregivers. The interview date was chosen by the participants or their family caregivers after considering their daily schedule (e.g., day-service program and nap time) and health condition.

Older adults were eligible to participate in this study if they were 95 years or older and capable of providing informed consent. In cases where a person had vision or motor function impairment, their family caregivers assisted in the consent procedure. Notably, we included individuals with cognitive impairments, provided they could answer questions. One of the 18 participants withdrew because of poor health; consequently, we interviewed 17 participants, all of whom provided written, informed consent.

### Interview guide

Based on a literature review of successful aging (Anderson, Goodman, Holtzman, Posner, & Northridge, 2012) and living experience (Fleming, Farquhar, Brayne, & Barcley; Cambridge city over-75s cohort (CC75C) study collaboration, 2016; Jopp et al., 2016; Wong et al., 2014) of older people, we developed a semi-structured interview guide that included positive (e.g., a feeling of happiness) and negative (e.g., physical and cognitive decline) aspects of their experience (Table I). Furthermore, the interview guide consisted of past, present, and future axes to facilitate the oldest old in talking freely. Some adjustments were made to the guide after the 4th, 8th, and 13th interviews, refining questions to facilitate conversation. Table I presents the final version of the interview guide.

**Table I.** Semi-structured interview guide.

How do you spend everyday life?
● What was the most difficult thing or event you experienced in your life?
● What are you proud of?
● What are the most important beliefs you have concerning life?
● Are you satisfied with your life?
● What decisions do you make on a daily basis, in your current situation?
● As you age, what are you no longer concerned about? What is important to you?
● From what do you obtain everyday pleasure?
● Have you lived your life as you wished?
● What do you think about death?

### Data collection

From July to November 2017, one of two investigators (HK or KY) visited the participants’ homes or nursing homes with a clinical psychologist or psychiatrist and conducted in-depth interviews, until saturation, when no new themes were emerging from analysis. To minimize the impact of each interviewer’s perspectives and increase the study’s credibility, a clinical psychologist or psychiatrist of the research team also attended the interviews. The investigators had no clinical relationships with any of the participants. Considering the frailty of the individual participants and the burden on family caregivers, we decided that only one interview was feasible and chose the most convenient date for the participants or their family caregivers.

The individual interviews were mainly conducted by experienced nurses (HK and KY) using the interview guide. The interviews were audio recorded with the permission of the participants and/or family caregivers. The interviewers kept a reflexivity journal that included observational and methodological notes and analytic memos after each interview. We also conducted a peer debriefing to share challenges in the research process and findings.

### Data analysis

We used the qualitative research software NVivo10® to record and manage the data. After all the interview data were transcribed, the transcripts were analyzed following the principles of thematic analysis (Braun & Clarke, 2006) (thematic analysis reveals the content of and meanings behind patterns (themes) present across entire data sets). The investigators (HK and KY) reviewed the transcripts several times, anonymizing personally identifiable information.

Adhering to the phases of thematic analysis (Braun & Clarke, 2006), the following procedures were implemented: (1) KY and HK independently, and continuously, reviewed the transcripts; (2) data that were determined to be relevant were extracted from the entire data set, and labelled with initial codes; (3) candidate themes were identified by repeatedly comparing and integrating individual codes and reporting patterns in the data; (4) themes were identified by reviewing the candidate themes; (5) subthemes were determined; and (6) quotations were selected for use in the results.

To ensure the trustworthiness of the study, we adopted the following procedures. One of the investigators (KY) analyzed the data, and her subjective perceptions were described in brackets. Then, another investigator (HK) reviewed the data with the initial codes and tentative subthemes. All the themes and subthemes were discussed, and final themes were established by KY and HK. Peer debriefing was conducted 3 times among the research team members to interpret the results. In consideration of the participants’ difficulties due to their functional decline in vision and hearing, we did not conduct member checking. After completing the identification of themes and quotations that supported the themes, a professional translator translated them into English.

### Ethical approval

This study was approved by the Institutional Review Board of the Keio University School of Medicine (No. 2015062). Further, written and oral informed consent was obtained from all participants or their family caregivers.

## Results

Seventeen participants (6 men and 11 women) participated in this study (Table II), with a mean age of 98.6 years (range: 97–103). Of these, 13 participants lived in their homes, with 6 living alone, and 4 stayed in nursing homes (Table II). As their concentration time was limited, the mean time of the individual interviews was short (37.5 min; range 15–65). With the exception of 4 interviews, 1 or 2 relatives were present for each interview.

**Table II.** Participant characteristics.

Mean age (SD), y	98.6 (1.68)
Gender	N
Men	6
Women	11
Living Arrangements	
Living alone	6
Living with a spouse and/or family	7
Nursing home	4
Level of Dependency	
Independent	4
Support level (almost independent)	2
Care level 1 (requiring partial assistance for housework)	0
Care level 2 (requiring partial assistance for toileting)	3
Care level 3 (requiring almost full assistance)	2
Care level 4 (requiring full assistance)	1
Care level 5 (totally dependent, with communication difficulties)	2
Unknown	3

Level of dependency was determined by consulting an assessment concerning eligibility for long-term care insurance.

Thematic analysis revealed 3 major themes, which also contained subthemes (Table III); these are presented herein and illustrated using representative quotes. We used the letters A through Q for the participants and described their age and gender in brackets after the quotations in italics.

**Table III.** Themes and subthemes.

**Unshakable beliefs and social ties**
Diligence and compassion
Strong ties with small networks
Hopes for younger generations
**Natural acceptance**
Acceptance of self
Acceptance of life
**My day-to-day life with precious moments**
One more routine day
Perceived control of life
Precious moments in life

### Theme 1: unshakable beliefs and social ties

#### Diligence and compassion

The participants retained strong belief in diligence and compassion. In their long lives they had experienced many changes, including war. They had overcome hardships in life with diligence or hardship and often mentioned diligence as a foundation for life: “*Just focus on living. That’s my opinion”(98-year-old woman, D)*. One participant described living this way as the secret to longevity: *“If you live sincerely, you can live for a long time” (101-year-old man, B).*

As the participants had gone through difficult situations, they understood the emotional state of another person and valued showing consideration to others: *“Life is built on shared warm-heartedness. Compassion is essential, regardless of whether you are young or old” (98-year-old woman, D)*. They said that they always tried to help others: *‘To date, I have done nothing wrong. I mean I have tried to do good things for others’ (98-year-old woman, M)*. Through their experience, they learned that people care about and help each other. Consequently, they accepted their situations as *“my turn to be taken cared for”: ‘we should never forget that helping others helps ourselves’ (97-year-old woman, J).*

#### Strong ties with small networks

The participants maintained strong ties with a limited number of people. In the past, they all had active interactions with their friends, but few of these friends were still living: *“I had many friends, but they are all gone” (97-year-old man, H)*. Another participant was nostalgic for the past: *“I exchanged New Year’s cards with nearly 300 people, but now I only have my family members and my younger brother and sister. No more. Yes, I miss them” (98-year-old man, L)*. All female participants had lost their husbands, and 1 had even lost her children. She lamented their deaths: “*My children died in their 60s. My 2 sons”(99-year-old woman, C).*

The participants’ frequency of interactions with others was high. Although some participants lived alone, their children visited them almost daily. The participants felt secure and happy when they had regular contact with familiar people: *“I am happy with them (many factory workers, who feel like my family). I am glad to hear them ask ‘How are you?’” (97-year-old woman, J)*. They also expressed deep gratitude for their family members: *“Now, I am thankful for my children. My older children help me; they are in their 70s. I am always grateful [for their help]” (101-year-old woman, F)*. Most participants expressed sincere affection for their family members: *“Yes, each member of my family is close to my heart” (100-year-old man, E)*. Family bonds created happiness: *“My children are my treasures. They make me happy” (97-year-old man, H).*

As a result of the decline in their functions, social participation was difficult for most participants. However, a small number of healthy older adults were still engaged in group activities, including hobby and senior clubs. One participant was committed to encouraging the frail members of his senior club to keep active: *“It may be an overstatement if I say I do it for others […] I try to include other members and think of ways of helping everyone to participate” (98-year-old man, L)*.

#### Hopes for younger generations

Although the objective social networks of the oldest old were small, their subjective social connectedness was generally strong. When they were asked about their hopes for the future, multiple participants mentioned the impact of war on younger generations. War was the most memorable negative event in their lives: *“[The war was] an accumulation of difficult experiences. We must not experience it again” (98-year-old woman, D)*. The participants hoped that younger generations would not have the same tragic experiences: *“I don’t want anybody to go through what we went through” (101-year-old woman, F)*. The oldest old were not only taken care of but they also cared about younger people. They were still members of society and their hopes were not about themselves but the future of younger generations.

### Theme 2: natural acceptance

#### Acceptance of self

The participants genuinely acknowledged and accepted themselves as they aged. They recognized their limitations and embraced themselves the way they were. One participant mentioned that she no longer desired to change herself: *“I no longer want to look good” (97-year-old woman, J)*. Another participant said that she no longer compared herself to others: *“Everything in moderation. There is always someone better or worse” (101-year-old woman, F)*. In fact, they became as positive about themselves as they were about others. One participant said she was formerly short-tempered but had changed: “*I realized that I haven’t gotten angry since I turned 80” (99-year-old woman, G)*. Self-acceptance led to accepting others.

#### Acceptance of life

During the course of their long lives, the participants had overcome innumerable bereavements and hardships. One participant who lost his wife said: *“Such is life” (98-year-old man, L)*. Some participants had lost their husbands and brothers in war, and 1 of the female participants raised her daughter by herself after a very short marriage. One male participant who was detained in Siberia during WWII said: *“My war experience made me believe that I could overcome any hardship and survive” (101-year-old man, B)*. Another participant accepted difficulties as unavoidable: *“I think [difficulty in life] is natural” (97-year-old man, A)*. Overall, most participants were satisfied with their lives: *“I have lived an ordinary life, but when I look back, it was not bad” (98-year-old man, L)*. Another participant said: *“I had difficulties during the first half of my life, but I was rewarded; I think the latter half has been joyful and good, so I am happy” (99-year-old woman, G).*

The participants did not expect much benefit from medicine: *“Visiting a doctor does not help me much” (101-year-old man, B)*. Another participant did not want to replace the battery of her pacemaker because she would live too long and was worried that she would bother other people. *“I am poorly educated, but Doctor, I don’t want to have it (pacemaker)…I was told I have to replace it (battery). But I will live too long and bother other people. So, I asked him, ‘Doctor, I wonder if I have to. Would you tell me?’ My hospital doctor said, ”You will be alright. You have to replace it.”” (97-year-old woman, O)*. They felt that death is also a part of life: *“We all die eventually” (97-year-old woman, O)*. Most participants did not think about living or dying, instead allowing nature to take its course: *“I don’t think about anything, I just continue. I do not think about living or dying” (100-year-old man, E)*. They were prepared to die; however, some were concerned about the impact it would have on their families. Another participant hoped that she would be missed: *“Whether I will be missed makes a difference to me” (97-year-old woman, J).*

### Theme 3: my day-to-day life with precious moments

#### One more routine day

The participants did not have future plans but lived each day naturally: *“At my age, I have nothing to aim for. I just wish to be my natural self” (101-year-old woman, F)*. Almost all participants preferred to continue living: *“I just want to continue to live peacefully with my son” (97-year-old woman, I)*. The participants’ lives were not complicated. They just followed simple daily routines: *“To live each day” (100-year-old man, E)*. Daily routines helped them lead a peaceful and happy life. The participants knew that life is tough, but they could make it easier. Their message for us was: *“Take it easy” (97-year-old woman, O).*

#### Perceived control of life

Living with restrictions may compromise autonomy. One participant expressed his frustration in this regard: *“I don’t like to be told what to do by others. I do as I like” (98-year-old-man, N)*. Most participants needed some help in regard to daily living, but they were not totally dependent on others. The small decisions they made, helped them feel that they still had a place in everyday life. One participant was looking forward to moving to the next room, which would allow her to see her family in the morning; it was her decision as to whether she saw her family or not. The ultimate example is 1 bed-bound participant. He expressed his satisfaction as follows: *“I appreciate that in this room I can sleep and wake as I like” (97 year-old-man, H).*

One participant had lost her house because of the construction of a new road, and she moved into a nursing home because she had no other choice. As she was aware that her small room would be the last place she would live, she reconstructed her environment using her imagination. She said: *“The beds are like houses in a neighbourhood. I lost my house, but this is my home, with a curtain (as a door). They [her roommates] are all my neighbours […] It’s OK” (98-year-old woman, P)*. She accepted her harsh reality and used her freedom of imagination to maintain her autonomy.

#### Precious moments in life

Although the activities the participants could perform were declining, they still managed to find joy in life. One woman who used a wheelchair was excited about mornings: *“I look forward to going to the Santoku [supermarket]” (103-year-old woman, Q)*. That was her routine, and she found enjoyment in it.

As the participants were approaching the end of their lives, they were living moment-to-moment. Small things that often to unnoticed mattered to them. One woman described a moment in her life she cherished: *“When the flowers bloom, it makes me happy” (99-year-old woman, C)*. Further, 1 participant, who was almost blind, felt that sunrises welcomed her to a new day, and this encouraged her to continue living a little longer: *“[Sunrises] help me feel that I can still see. Because my eyesight is deteriorating, I am happy if I can see anything. It makes me believe that I can make it through 1 more day” (97-year-old woman, I)*. Appreciating these small pleasures made them happy and feel that life was worth living.

## Discussion

This study determined that the oldest old maintain their individual beliefs and social ties, accept their selves and their lives, and live each day with perceived control of their lives and finding pleasures in simple moments.

People make decisions and behave based on their beliefs. The oldest old in this study believed that rewards come from hard work and that if you “do good for others, it will return to you.” In Hong Kong, another super-aging society, near-centenarians and centenarians provided similar advice to younger people: that longevity is largely related to attitude towards life (Wong et al., 2014).’ Further, showing concern for others strengthens interdependence; interdependence, in terms of relational harmony, along with independence, defined as personal control, have been found to enhance health and wellbeing (Kitayama, Karasawa, Curhan, Ryff, & Markus, 2010).

Social connectedness is important for overall quality of life (Rogers & Mitzner, 2017). Although the networks of the oldest old were small, their ties with family members, professionals, friends, and neighbours were strong because of regular contact. Such regular contact provides opportunities for social exchange with a small, yet regularly present, group of informal and formal caregivers (Jopp et al., 2016). Despite their small networks, the oldest old felt they had a moral obligation to contribute to society. Since historical events had greatly affected their lives, they strongly hoped that younger generations would make better decisions: no more wars. The oldest old not only care about younger people, but also society, and they want to bequeath the wisdom they have obtained to younger generations.

A long life has both positive and negative aspects. Although age-associated functional and social losses are inevitable side-effects of exceptional survival, the oldest old have a high ability to adapt to these changes (Darviri et al., 2009; Molton & Yorkston, 2017). Further, their views on death are consistent with those reported in related studies; they are not afraid of dying (Darviri et al., 2009; Fleming et al., 2016), but have concerns about the impact their deaths will have on their families and carers (Fleming et al., 2016). These people also tend to avoid having any conflicts in regard to relationships and do not wish to compare themselves to others (Darviri et al., 2009) when the oldest old accept themselves and their lives, both the good and the bad, they become comfortable being themselves, and find peace and happiness. Consequently, the oldest old are mostly satisfied with their present lives (Darviri et al., 2009; Eloranta et al., 2012; Zeng et al., 2017), and their happiness is not influenced by their physical limitations (Jopp et al., 2016). Analysis of the developmental course of emotional experience has revealed that as people age, overall emotional well-being becomes more positive and stable, and that individuals who are positive are more likely to live longer than those who commonly feel negative emotions (Carstensen et al., 2011).

Control over personal decisions is central to human dignity (Lette et al., 2017; Molton & Yorkston, 2017). The oldest old’s sense of control is improved by being allowed to do the things they are still capable of doing, but a previous study has suggested that there are discrepancies between the perspectives of older adults and those of health-care professionals in this regard: while older adults prefer to be autonomous and self-sufficient, professionals are likely to intervene at an early stage and curtail this autonomy (Lette et al., 2017). One of the participants stated he did not like to be given instructions regarding his daily life; this represents his desire for autonomy. The involvement of other people prevented him from making choices that accorded with his values and preferences, including how he was supported. Autonomy does not mean independence autonomy means the ability to exercise choices (Molton & Yorkston, 2017). Although for the oldest old exercising autonomy means often making very small decisions, such decisions are important to them, and make them feel that life is worth living.

The oldest old do not have future plans, instead focusing on the present; that is, “living for today (Eloranta et al., 2012; Wong et al., 2014).” For such individuals, who have limited life expectancy, the beauty of life is present in simple moments. A previous study has shown that older adults have a strong attachment to places; they feel very positive about their homes and the comfort they feel there, and they also enjoy the sunshine and their gardens (Wiles et al., 2009). A sense of belonging or attachment to a place may help maintain a sense of identity and wellbeing, and facilitate successful adjustments in old age (Wiles et al., 2009).

The limits of medicine are a focus of attention in aging countries. Disease- and problem-oriented approaches are necessary to address this, but these require improved medical expertise, and are hampered by the fact that many physicians struggle to balance their own beliefs regarding appropriate treatment with respecting the autonomy of older adults (Zwijsen, Nieuwenhuizen, Maarsingh, Depla, & Hertogh, 2016). Nevertheless, in future systems the aggressive care provided for certain medical conditions could be reduced (Arai et al., 2015), and more appropriate treatment for older adults that reflects the realities of how they live their lives could be implemented. Our findings demonstrate that very small things that are often unnoticed are significant for the oldest old, and address what they mean by their freedom of choice and pleasures in life.

### Limitations

This study has several limitations. First, the size of our study sample was small. All participants in this study were relatively healthy and functional. However, other oldest old individuals with different conditions may have different perspectives. Thus, these results may not be broadly generalizable to larger populations. Second, it was extremely difficult to conduct the interviews. As a result of their limited concentration, long interviews were not feasible. In addition, many participants had hearing impairments or lower cognitive function, so the interviewer was forced to speak louder and closer to the participants or repeat questions. Third, since their daily lives were so normal to them and nothing was special, it was difficult for them to talk about themselves in great detail. Fourth, although multiple interviews are important to obtain rich data, we conducted only one interview for each participant in consideration of the participants’ frailty and the burden on their old family caregivers (70−90 years).

On the other hand, many participants were very pleased that their participation in the study could contribute to assisting younger generations. Their family members were also satisfied with the interviews because they gained an understanding of their parents’ feelings and how they have lived, and they reacquired their affection and gratitude for them.

## Conclusions

This study represents an important step towards developing specific health policies for an aging population. The oldest old want to continue their peaceful routine days, maintaining their firm beliefs and strong ties with their small social networks. While they are accepting of their present situations, making small choices is important for these people to perceive control of their lives, and finding small pleasures in simple moments makes their lives worth living. Considering our findings, it can be suggested that policy makers and healthcare providers avoid depriving the oldest old of these simple moments, and instead help them to achieve that which is important to them.
